# Effects of Palm Oil Nanoparticles in Diverse Physical States on the Properties of Starch Films

**DOI:** 10.3390/foods15010139

**Published:** 2026-01-02

**Authors:** Yaqi Zhang, Qianwen Yang, Zhao Li, Qingqing Chai, Zheng Zhang, Na Wang, Lu Lu, Meng Zhao, Bo Cui

**Affiliations:** State Key Laboratory of Green Papermaking and Resource Recycling, Shandong Key Laboratory of Healthy Food Resources Exploration and Creation, School of Food Science and Technology, Qilu University of Technology, Shandong Academy of Sciences, Jinan 250353, China; zhangyaqi202511@163.com (Y.Z.); yqw15716333915@163.com (Q.Y.); 1z8802020186@hotmail.com (Z.L.); qqchai890321@163.com (Q.C.); zhengzhang324@163.com (Z.Z.); rizhaowangna1222@163.com (N.W.); lulu19861117@126.com (L.L.)

**Keywords:** nanoparticles, palm oil, mechanical properties, barrier properties, thermal properties

## Abstract

Most previous research focuses on single-state palm oil (PO) modification of starch films, while the interaction between different physical states PO and starch matrix has not been deeply discussed. This study aimed to investigate the effects of PO nanoparticles in three physical states (liquid, semi-solid, solid) on starch-based films, where the physical state of PO nanoparticles was regulated by manipulating the melting point of PO. PO nanoparticles with five different melting points (8, 24, 33, 42, and 53 °C) were prepared at 30 °C using emulsification with sodium caseinate as the emulsifier and were integrated within a starch matrix to fabricate films. The findings revealed that the starch film with 33 °C PO nanoparticles had the smoothest and most homogeneous surface, the best dispersion state of the oils, the optimal compatibility, and the highest film crystallinity. These films exhibited enhanced tensile strength (TS), stiffness, and barrier properties. Furthermore, starch films containing solid nanoparticles exhibited superior thermal stability. This study innovatively prepared nano-scale palm oil-starch composite films and revealed the pivotal role of the viscoelastic attributes of semi-solid PO nanoparticles in enhancing the qualities of starch-based films.

## 1. Introduction

Amidst the heightened awareness of environmental preservation and the deepening of the sustainable development concept, the field of food packaging materials is undergoing a profound transformation. Starch is gaining significant attention for edible film and packaging applications due to its favorable biocompatibility and biodegradability [1]. However, the inadequate water barrier properties of traditional starch-based films limit their wide application in different environments. To tackle this constraint, researchers have investigated the incorporation of hydrophobic components into starch-based films [2,3,4,5]. Among the numerous hydrophobic substances, vegetable oils are favored for their accessibility, renewability, non-toxicity and non-volatility. Moreover, their rich content of unsaturated fatty acids suggests potential health benefits when incorporated into edible films or coatings [6].

Due to the hydrophilicity of starch, the compatibility of the vegetable oil component with the starch substrate is also a key point that affects the performance of the composite film. Palm oil (PO) could be a potential candidate [7]. Previous research has reported that the incorporation of PO reduces the WVP of gelatin films [8]. The smaller the size of the PO droplets, the more uniformly they were distributed across the film [9]. Therefore, in this study, PO was emulsified into nanoparticles, which effectively solved the compatibility problem of the two-phase interface. For clarity of presentation, the droplets in nanoemulsions are collectively referred to as nanoparticles in this paper.

PO exhibits different states at room temperature due to variations in its melting points. Based on these melting points, POs can be classified into types such as 8, 24, 33, 42, and 53 °C. PO with different melting points can be processed into nanoparticles in distinct physical states. Sodium caseinate (CAS) has been proven to be an excellent emulsifier [10], effectively improving the distribution of oil in films. To our knowledge, systematic research exploring the impact of PO nanoparticles physical state on starch film properties remains scarce.

The addition of soft metal particles to ceramic-based films has been reported to significantly enhance the mechanical properties of the films, compared to the addition of hard metal particles [11]. When compared to the incorporation of three liquid oil emulsions (palm oil, virgin coconut oil, and canola oil), the addition of semi-solid emulsion (cocoa butter replacer) into soy protein isolate gel yielded gels with the highest hardness and gel strength [12]. Therefore, the addition of palm oil nanoparticles may enhance the mechanical properties of starch-based films, and films containing semi-solid palm oil nanoparticles may have better performance than those containing liquid and solid palm oil nanoparticles. The aim of this research endeavor was to undertake a comprehensive investigation into the effects of PO nanoparticles in different physical states (liquid, semi-solid, solid) on the properties of starch films. The physical states of the nanoparticles were regulated by the melting points of PO and five POs with the melting points of 8, 24, 33, 42, and 53 °C were used. And the palm oil was made into nanoparticles by the emulsification method using CAS as an emulsifier, aiming to enhance its compatibility with the starch matrix.

## 2. Materials and Methods

### 2.1. Material

Corn starch was sourced from Macklin Biochemical Co., Ltd. (Shanghai, China). Sodium caseinate (CAS, product number C0594) from Tokyo Chemical Industry Co., Ltd. (TCI, Tokyo, Japan). Glycerol was purchased from Tianjin Fuyu Chemical Co., Ltd. (Tianjin, China). POs with different melting points (8, 24, 33, 42, and 53 °C) were purchased from Qinhuangdao Jinhai Grain and Oil Industry Co., Ltd. (Qinhuangdao, China).

### 2.2. Preparation and Characterization of PO-Nanoemulsions with Different Melting Points

The CAS powder was dispersed in Milli-Q water for a duration of 4 h at a temperature of 45 °C to prepare a 0.25% stock solution. POs with distinct melting points were mixed with the CAS solution at a concentration of 1% (*w*/*v*), and this mixture was homogenized using a Fluko FA25 homogenizer at a speed of 10,000 rpm for 2 min (Fluko Equipment Shanghai Co., Ltd., Shanghai, China). Subsequently, a 25 g portion of the emulsion was subjected to ultrasonication using a titanium probe with a diameter of 0.600 cm. The ultrasonic power applied was 400 watts, and the process lasted for 6 min, with a cycle of 2 s on and 2 s off.

The particle size distribution and degree of dispersion (Ð) of the nanoemulsions were evaluated at 30 °C using a Zetasizer Nano-ZS (Malvern Instrument Co., Ltd., Worcestershire, UK). The prepared nanoemulsion was diluted about 100 times with Milli-Q water.

### 2.3. Preparation of Starch–PO Films

A total of 2.5 g of corn starch was dissolved in 25 mL of Milli-Q water. The film-forming solution included the following: 10% corn starch suspension, glycerol of 0.7 g, and CAS-PO nanoemulsions prepared in Section 2.2. After 30 min of heating to 90 °C and degassing with a filter cloth, the mixtures were poured into Petri dishes with a diameter of 15 cm. The drying process lasted 24 h at 30 °C. Once the drying process was completed, the final starch–PO film was carefully peeled off. This film was then allowed to equilibrate at 25 °C and RH of 60% for 48 h, in preparation for further analysis or investigation. Six distinct groups of starch–PO films were prepared, each designated with a specific label: control, PO8, PO24, PO33, PO42, and PO53 were subjected to measurements. The specific compositions are shown in Table 1.

### 2.4. Rheology of Film-Forming Solutions

The ungelatinized film-forming solution mixture samples prepared in Section 2.3 were subjected to rheological analysis. At a fixed temperature of 30 °C, all samples were evaluated for their rheological properties using an MCR-302 rheometer (Anton Paar GmbH, Graz, Austria). Viscosity (η*) was measured in the frequency range of 0.01–100 rad/s [13].

### 2.5. Color Parameters

The color parameters of the starch–PO films (including L^*^, a^*^, b^*^ and Δ*E* values) were measured using an ADCI-60-C Colorimeter (Chen-taike Instrument Co., Ltd., Beijing, China) [14]. The measurement aperture is 8 mm, the light source used is the D65 standard light source, and the observer type is a 10-degree field of view. The colorimeter was used to measure the film. At least three points were randomly selected from each sample to estimate the average value. The color parameters for the background of the films were established based on the color characteristics of the control group.

The following equation is used to calculate the Δ*E* value of the starch film to estimate the color difference between the samples [9].

$$\Delta E=\sqrt{{\left({L_{0}}^{*}-L^{*}\right)}^{2}+{\left({a_{0}}^{*}-a^{*}\right)}^{2}+{\left({b_{0}}^{*}-b^{*}\right)}^{2}}$$

where L^*^, a^*^ and b^*^ are the color parameter of Control (*L*_0_^*^ = 53.93, *a*_0_^*^ = 1.81, *b*_0_^*^ = 2.24) used as the film background.

### 2.6. Thickness, Moisture Content, Total Soluble Matter Content and WVP of Starch–PO Films

#### 2.6.1. Thickness

The thickness was assessed by an MDC-25PX Digimatic Micrometer (Mitutoyo Corporation, Tokyo, Japan). Ten randomly selected points from each sample were used to estimate the mean value.

#### 2.6.2. Moisture Content and Total Soluble Matter Content

Initially, the film samples, each with dimensions of 20 mm × 20 mm, were weighed (m_1_), and then reweighed (m_2_) after drying at 110 °C for 24 h. 30 mL of Milli-Q water was introduced to the equilibrated film samples (20 mm × 20 mm), which were then subjected to vortex mixing at 180 rpm for 24 h at 25 °C. Following this, the samples were dried at 110 °C for an additional 24 h. (final dry mass = m_3_) [15]. The following was the formula:

Moisture content (%) = (m_1_ − m_2_)/m_1_ × 100

Total soluble matter content (%) = (m_2_ − m_3_)/m_2_ × 100

#### 2.6.3. WVP

Values of WVP were calculated using a previously published method [16]. The Water Vapor Transmission Rate Tester (PERME™ W3/030, Lab Think Instruments Co., Jinan, China) was used. The samples, cut into circles with a diameter of 6.48 cm, were positioned within the instrument and subjected to a 10 h measurement at 38 °C and a relative humidity of 90%. The average WVP values were determined from three different samples.

### 2.7. Mechanical Properties of Starch–PO Films

Tensile strength (TS) and elongation at break (EAB) of starch–PO films were analyzed using an automatic tensile testing machine (Param Xlw, Jinan, China) [10]. The sample films were cut into strips measuring 70 mm × 10 mm, and each test was performed at a crosshead speed of 50 mm/min. A minimum of five repetitions for each film were tested.

The measurement parameters utilized were as follows: 5 µm amplitude, 1 Hz frequency, −100 °C to 150 °C temperature range, and 5 °C/min heating rate.

### 2.8. Structural Characteristics of Starch–PO Films

#### 2.8.1. Microstructure Observation of Starch–PO Films

Film samples were affixed to the sample stage using conductive tape and observed at 100× magnification for surface characteristics and 500× for cross-sectional details.

A total of 25 μL (0.1%, *w*/*v*) of Nile Red solution was added to 50 g of the film-forming mixture for PO staining. The films were prepared as described in Section 2.3. Throughout the procedure, strict light was avoided during the measurement. The film samples were stained and observed under 40× magnification, with images acquired by stimulating Nile red fluorescence using a 488 nm laser. The imaging covers a 25 μm range and a 1 μm step size [10].

#### 2.8.2. Fourier Transform Infrared Spectroscopy (FTIR) of Starch–PO Films

Measurements were conducted in ATR mode with 32 scans, covering a wavenumber range of 4000–500 cm^−1^, with a resolution of 4 cm^−1^.

#### 2.8.3. X-Ray Diffraction (XRD) of PO and Starch–PO Films

Crystallization parameters were analyzed using an X-ray diffractometer (SmartLab SE, Rigaku, Tokyo, Japan) operated at 40 kV and 40 mA. PO samples with different melting points were melted and measured at 30 °C. The PO samples and starch–PO film samples underwent diffraction at a scanning rate of 20°/min, with the diffraction angle varying between 4° and 40°.

### 2.9. Thermogravimetry (TG) Analysis of Starch–PO Films

An STA 6000 simultaneous thermal analyzer (Perkin Elmer, Shelton, WA, USA) was used for the TG analysis. Accurately weigh the film samples (10 mg) and conduct the tests within a temperature range of 40–600 °C at a heating rate of 10 °C/min.

### 2.10. Statistical Analyses

Statistical analysis was performed using SPSS version 26.0 (SPSS Inc., Chicago, IL, USA), and data processing was conducted with Excel 2019. Significant differences in the mechanical, physical, and optical properties of the films were assessed using the Waller–Duncan test and ANOVA.

## 3. Results

### 3.1. Characteristics of PO with Different Melting Points

**Figure 1:**
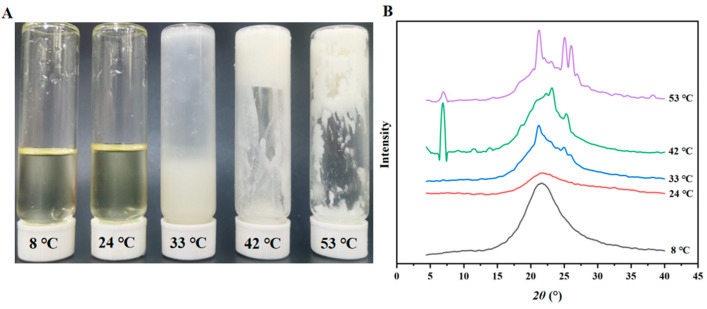
(**A**) The appearance and (**B**) XRD patterns of POs with different melting points at 30 °C.

As shown in Figure 1A, POs with different melting points exhibited distinct physical states at 30 °C. POs with melting points of 8 and 24 °C were close to liquid; those with melting points of 42 and 53 °C were nearly solid, while PO with a melting point of 33 °C exhibited an intermediate transitional state, displaying a flowable yet viscous consistency, and thus was defined as a semi-solid phase. Since the state of PO varied from one melting point to another, the state of the above PO emulsion was different. Especially, the nanoemulsions of 8 and 24 °C PO corresponded to liquid nanoparticles, the nanoemulsion of 33 °C PO corresponded to semi-solid nanoparticles, and the nanoemulsions of 42 and 53 °C PO corresponded to solid nanoparticles.

In the XRD pattern presented in Figure 1B, the analysis of PO at different melting points at 30 °C revealed a characteristic feature: the liquid oil region did not exhibit any significant diffraction peaks, suggesting that the liquid oil contained little detectable crystalline content at this temperature. In contrast, weak diffraction peaks appeared in the XRD pattern of 33 °C PO, suggesting the presence of a minor crystalline component in PO. This corresponds to the semi-solid state of 33 °C PO shown in Figure 1A. Further, the XRD patterns of the solid oil showed sharper and clearer diffraction peaks, indicating that the molecules within the PO were more organized. This led to the development of a more pronounced crystalline structure. Therefore, it could be deduced that the crystallinity of PO at 30 °C increased significantly with the increase in the PO melting point, and its physical state gradually transitioned from liquid to semi-solid and finally solid, a process accompanied by the gradual establishment and improvement of the crystal structure.

### 3.2. Nanoemulsions Properties

Table 2 presents the mean diameter and polydispersity index of PO nanoemulsions with different melting points. Observations indicated that the two solid nanoemulsions (PO42 and PO53) exhibited comparable average diameters, both higher than those of the liquid formulations (PO8 and PO24) and the semi-solid nanoemulsion (PO33). PO8 and PO33 demonstrated similar average diameters, which were smaller than PO24, with PO33 showing the smallest average diameters among all samples, whereas a larger diameter suggested a larger droplet volume in the emulsion and more severe aggregation [9]. Although no statistically significant differences were detected in the polydispersity index (PDI) among PO8, PO24, and PO33, a gradual decreasing trend in PDI values was observed, with PO33 exhibiting the lowest PDI. This numerical trend may suggest the most homogeneous particle size distribution, i.e., the particles in the particle population were the closest in size to each other. Both average diameters and PDI responded that PO33 nanoemulsions were the most stable and less aggregated.

The average diameter of the droplets in nanoemulsions is intimately associated with the surfactant and PO used. Previous experiments have shown that the utilization of CAS and ultrasonication contribute to the formation of nanoemulsions [10]. Since the same emulsifier was used for all PO nanoemulsions with different melting points, the effect of emulsifier on the average diameter could be negligible. Therefore, in this study, the effect of PO properties on the average diameter was dominant. At the same temperature, the PO with higher melting point exhibited higher viscosity than that with lower melting point, but only a proper oil viscosity was favorable for emulsification and emulsion stability [17]. The 33 °C PO had the right viscosity, resulting in a small mean diameter. On the contrary, 42 °C and 53 °C POs were more resistant to droplet formation due to their higher viscosity, leading to larger average droplet diameters. 8 °C and 24 °C POs had lower viscosities and tended to turn into smaller droplets at high shear speeds. However, new interfaces were formed when the droplets were dispersed into smaller droplets. The tension at these interfaces tended to reduce the surface area, causing them to come closer and merge, which increased their mean diameter. The viscosity of PO also affects this process, increasing the average diameters. PO24 has greater viscosity than PO8. This makes its particles grow faster. As a result, the average diameter of PO24-nanoemulsion was greater than that of PO8-nanoemulsion in Table 2. Similar results were found in nanoemulsions, with their nanoemulsions exhibiting average diameter of about 3 μm [9].

### 3.3. Rheological Properties of Film-Forming Solution

**Figure 2:**
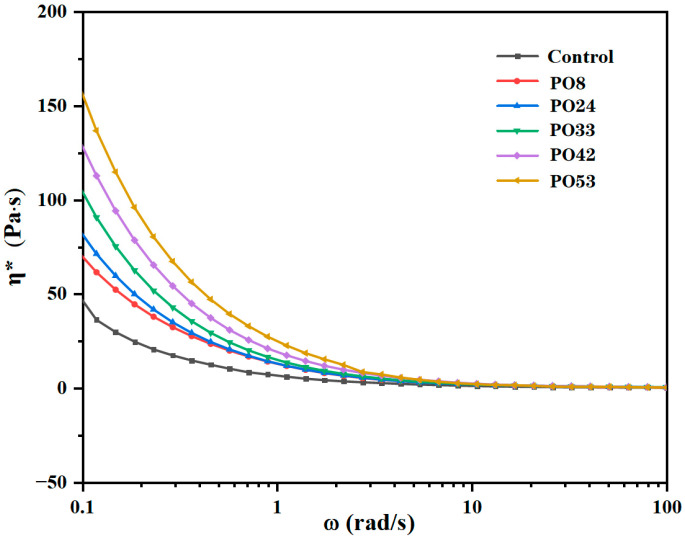
Apparent shear viscosity (η*) of film-forming solutions containing POs with different melting points at 30 °C.

Figure 2 shows the viscosity values of film-forming solutions containing PO nanoemulsions. From the viscosity profiles, all samples exhibited higher viscosity values compared to control. The viscosity of samples increased with the melting point of the added oil, with the highest viscosity observed in the PO53 solution and intermediate viscosity in the PO33 solution. Studies have shown that the optimum viscosity was a crucial determinant in the design and efficient production of food packaging films [18]. The viscosity of film-forming solutions partially affected the film properties, as shown in Section 3.5.2 and Section 3.7.

### 3.4. Optical Properties of Starch–PO Films

**Figure 3:**
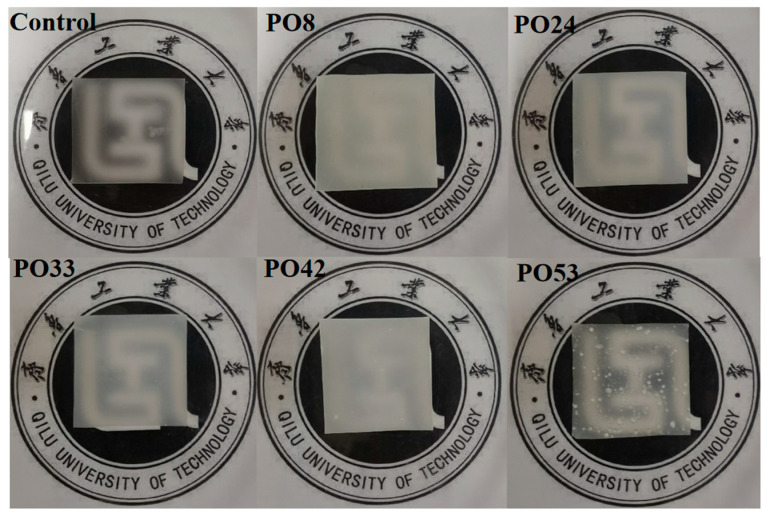
Appearance of starch–PO films containing POs with different melting points.

The color of the film affects the appearance of packaging materials and consumer acceptance. Figure 3 shows the appearance and light transmission of starch–PO films. Among them, PO33 was observed to have the clearest background pattern, indicating the best light transmission. PO53 film also demonstrated good light transmittance, but visible oily particles were observed on its surface. This phenomenon may correlate with the high crystallinity of 55 °C PO. In packaging applications, transparency is a big factor affecting the application of starch films, significantly impacting the readability of labels and the clarity of printed text [19].

Table 3 shows the L^*^ value, a^*^ value (red), b^*^ value (yellow), and ∆*E* of the films. The smallest L^*^ values for PO33 and PO53 may be due to the high crystallinity of PO33 and PO53 in Section 3.5.4. Crystallization reflected more lights and darkens the surface of the films. The a^*^ values of the films did not change significantly with the addition of PO nanoparticles. The b^*^ values of all experimental groups were higher than the control, which may be attributed to the natural pigments in palm oil inducing yellowing of the films. PO33 had the largest ∆*E*, which was significantly higher than the control, indicating the most significant difference in color from the control. It is possible that the addition of semi-solid PO nanoparticles significantly changed the microstructure or chemical composition of the films.

### 3.5. Structural Characteristics of Starch–PO Films

#### 3.5.1. SEM of Starch–PO Films

**Figure 4:**
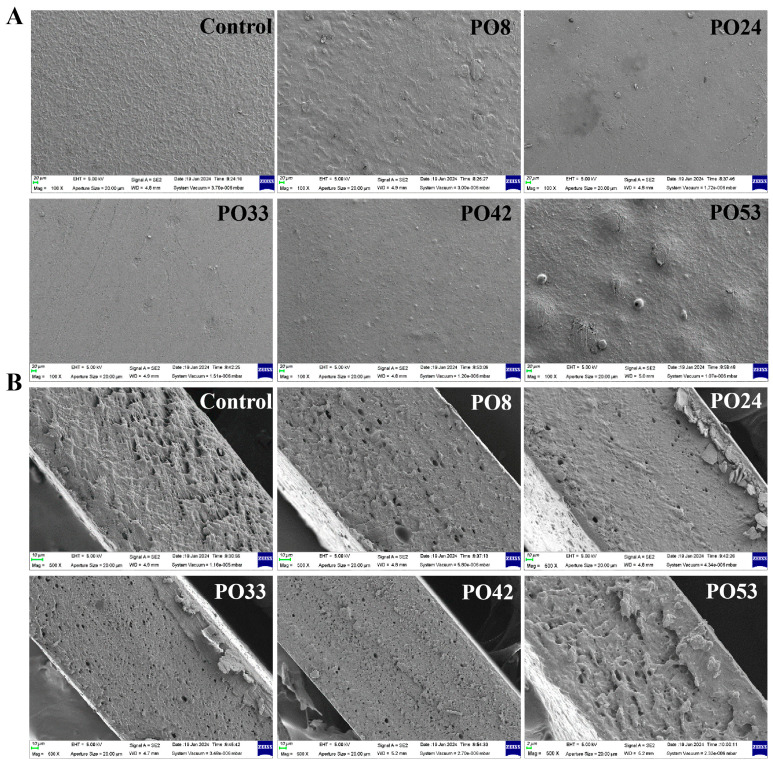
SEM of (**A**) surface 100× and (**B**) cross-section 500× of starch–PO films containing POs with different melting points.

Figure 4A illustrates that the control group displayed a uniform and non-porous surface, indicating corn starch’s superior film-forming capability. After the addition of PO nanoparticles, the surfaces of certain films exhibited roughness. The surface of the PO33 film was the smoothest and most uniform, potentially due to the best compatibility of PO33 semi-solid nanoparticles with the film substrate. In contrast, particles were observed in both surfaces of PO42 and PO53 films, with PO42 exhibiting significantly smaller particle sizes compared to PO53.

The cross-section of the PO33 film had small and uniformly distributed pores, whereas the cross-sections of PO42 and PO53 were rougher and showed aggregation of particles, particularly noticeable in the PO53 film (Figure 4B). These pores may result from oil aggregation and evaporation during the film formation process [20]. The PO33 film displayed a denser structure, likely due to the smallest particle size of the semi-solid PO nanoparticles. Previous studies have indicated that smaller oil particles tended to contribute to a more ordered and stable film network structure, whereas larger oil particles might introduce more disorder, affecting the overall film performance [21].

#### 3.5.2. CLSM of Starch–PO Films

**Figure 5:**
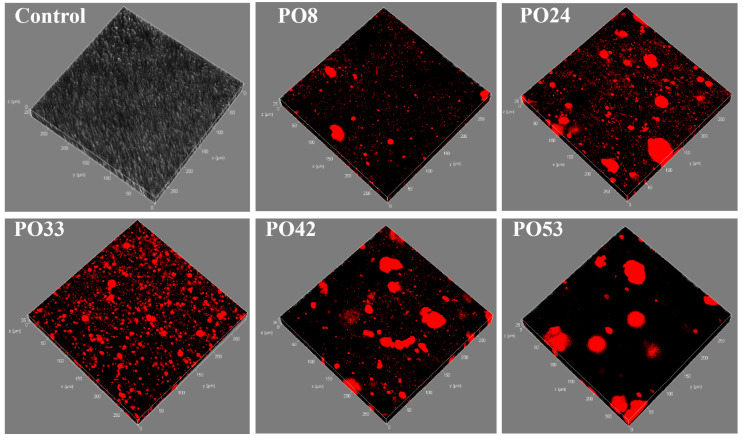
CLSM images of starch–PO films containing POs with different melting points (the red fluorescent signals are PO).

The semi-solid nanoparticle film of PO33 had the most uniform distribution of oil, exhibiting a small droplet morphology (Figure 5). In contrast, the oil in the liquid nanoparticle film of PO8 and PO24 was more likely to show large droplets, while the oil in the solid nanoparticle film of PO42 and PO53 tended to appear in lamellar states. In comparison, the number of oil droplets distributed on the film surface was ranked as follows: PO33 > PO24 > PO42 > PO8 > PO42. And both of the liquid and solid nanoparticle films exhibited uneven oil droplet distribution. This might be related to the physical properties of the nanoemulsions. Semi-solid PO, due to its proper viscosity, formed excellent dispersion within the film. This improvement in dispersion could be linked to the favorable compatibility between CAS, PO, and starch. Previous work reported that mixing the fibrous carboxymethyl cellulose (hard phase, film matrix) with spherical cellulose (soft phase, toughening agent) could improve the toughness of the whole cellulose film [22]. It has been reported that semi-solid shortening exhibited the smallest protrusions and the best dispersion state in agar/maltodextrin (A/M) films [23]. This finding aligned with our experimental results.

#### 3.5.3. FTIR of Starch–PO Films

**Figure 6:**
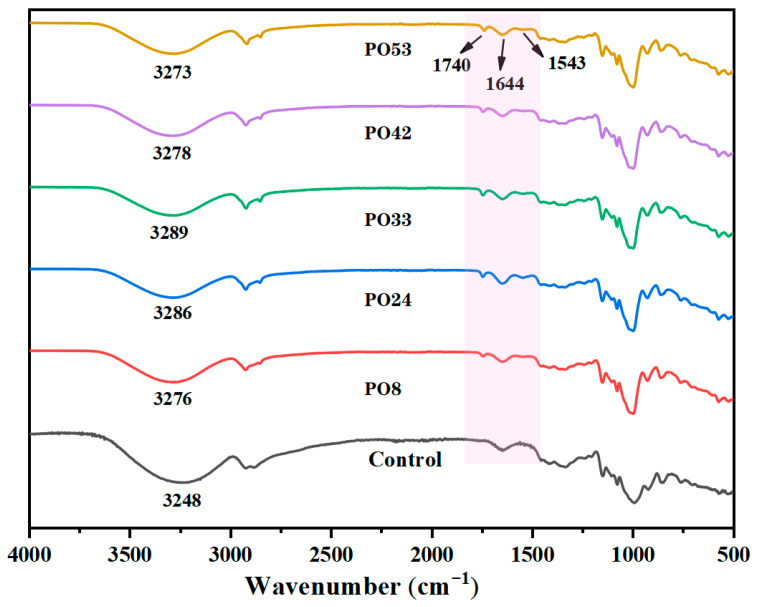
FTIR spectra of starch–PO films containing POs with different melting points.

All films exhibited a prominent peak at approximately 3248 cm^−1^ (Figure 6), likely attributed to extensive hydroxyl stretching vibrations present [24]. The characteristic peak of the PO nanoparticle films shifted to a higher wavenumber (Figure 6), suggesting the formation of hydrogen bonds (both intermolecular and intramolecular) between PO nanoparticles and corn starch. A new characteristic peak emerged at 1740 cm^−1^, which was associated with the addition of PO nanoparticles. This phenomenon may be attributed to the characteristic of the ester groups in the PO molecules absorbing infrared radiation through vibrational interactions. The characteristic peaks at 1644 cm^−1^ and 1543 cm^−1^ were related to the addition of CAS. The typical characteristic peaks of proteins are the strong amide I band and amide II band [25,26]. However, as observed in the figure, the peak at 1543 cm^−1^ was relatively small, which might be due to the lower amount of CAS added.

#### 3.5.4. XRD of Starch–PO Films

**Figure 7:**
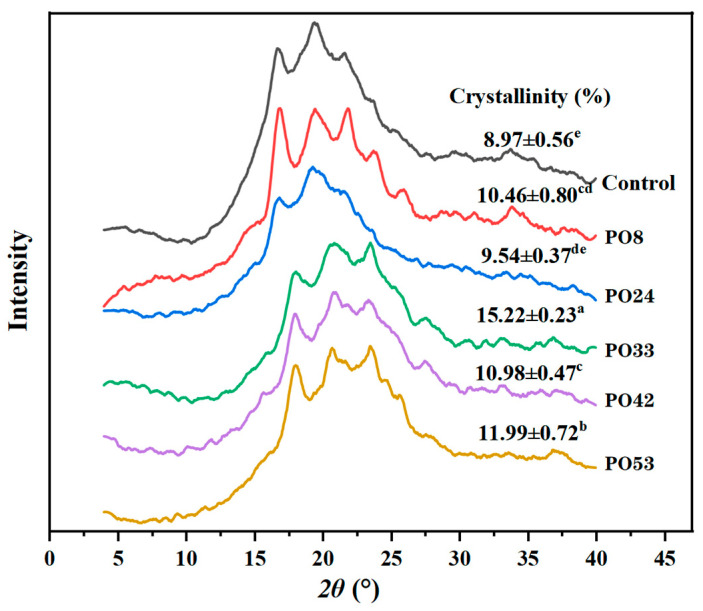
XRD patterns of starch–PO films containing POs with different melting points. Values with the same lower-case letter in a column are not statistically different (*p* > 0.05).

The control group exhibited diffraction peaks at 16.9°, 19.7°, and 21.9° (Figure 7). This was ascribed to gelatinized starch recrystallization [27]. PO nanoparticle incorporation resulted in enhanced film crystallinity. The order of crystallinity was: PO33 > PO53 > PO42 > PO8 > PO24 > control (Figure 7). The PO33 film, containing semi-solid PO nanoparticles, exhibited the highest crystallinity, which might be due to a tighter binding between the semi-solid PO nanoparticles and corn starch during film formation. In addition to the interaction between starch and oil, the crystallization of PO also influenced the crystallinity value of films. As shown in Figure 1, the crystallinity values of 42 °C and 53 °C POs were higher than those of 8 °C and 24 °C POs, explaining why the crystallinity values of PO53 and PO42 films were greater than that of PO8 and PO24 films. PO33 exhibited the maximum crystallinity value among the films. This could be due to the stronger interaction between semi-solid PO nanoparticle and starch. Previous research indicated that robust interactions resulted in a notable enhancement of crystallinity in chitosan-gum Arabic films, subsequently improving their mechanical properties [28].

### 3.6. Basic Properties of Starch–PO Films

Table 4 presents the thickness, WVP, moisture content and total soluble content of the starch–PO films.

#### 3.6.1. Thickness

The starch–PO films exhibited thickness values consistently greater than those of the control. This indicated that the added PO molecules prevented the interaction of starch chains from forming a dense mesh structure. The lowest value of thickness was found for PO33. This indicates that semi-solid PO nanoparticles had the least effect on the interaction of starch chain. This result aligned with the observation that the PO33 had a relatively flat surface in Figure 4A.

The thickness values of PO42 and PO53 were larger than that of PO33. This phenomenon may be due to the fact that PO, which has a higher melting point, also has a higher crystallinity, as shown in Figure 1. The interaction between starch molecules was interfered with crystals, leading to a looser film network, a more expanded film texture, and ultimately, an increase in thickness [9]. Furthermore, the viscosity of the film-forming solution also influenced the film thickness. As shown in Figure 2, the higher viscosity values of the PO42 and PO53 film-forming solutions resulted in poorer fluidity and thicker films. The thicknesses of the PO8 and PO24 were also larger than that of PO33, which may be due to the higher fluidity of liquid PO, increasing the probability of PO particle aggregation, leading to increased starch film thickness.

#### 3.6.2. WVP

The WVP values of the experimental films were significantly lower than those of the control group. In comparison, the films were ranked in order of their ability to block water: PO33 > PO24 > PO42 > PO8 > PO42. The poor barrier performance of starch films is attributed to their hydrophilic nature. The incorporation of hydrophobic substances can mitigate the hydrophilicity of the films. Moreover, the incorporated PO molecules create a tortuous pathway, hindering the penetration of water molecules through the film [29]. PO33 exhibited the lowest WVP, indicating its superior barrier properties. The reason for this could be that semi-solid PO nanoparticles had the highest dispersion, which facilitated the interaction between lipid molecules and the hydrophilic groups in the starch matrix and reduced the number of free hydroxyl groups. The lipid molecules could fill the free volume of the polymer matrix, creating a more compact structure and obstructing the diffusion path of water molecules [30], thus reducing the hygroscopicity of the film and the penetration ability of water molecules.

Another reason for the higher WVP values of PO8, PO24, PO42 and PO53 may be related to the average particle size of the nanoemulsions (Table 2). Semi-solid PO nanoparticles had the smallest average particle size, which facilitated their formation of more tortuous paths, thus better preventing water from passing through the film. This was consistent with the finding that the lower the average diameter of the stearic acid-treated film, the lower its WVP value [21]. While the average particle size of PO8 was smaller than PO24 and PO42, the WVP was higher than PO24 and PO42. The reason may be related to the distribution of nanoparticles on the film surface (Figure 5), and the ordering of the two sets of data was very consistent (PO33 > PO24 > PO42 > PO8 > PO42). PO8 had fewer palm oil nanoparticles distributed on the surface, which resulted in a water-blocking ability decreased.

#### 3.6.3. Moisture Content and Total Soluble Matter Content

The moisture content and total soluble matter content of films decreased after adding PO nanoparticles. The measured moisture content of PO33 was the lowest. Although no statistically significant differences were detected in total soluble matter content between the PO8, PO33, and PO42, it was observed that the soluble content of the PO33 group still exhibited the lowest value. This may be explained by the way PO nanoparticles interact with the hydroxyl groups in corn starch, which reduces the capacity of films to absorb water. The incorporation of hydrophobic substances decreased the hydrophilicity of the film, aligning with the findings of WVP. Among them, the largest decrease was observed in PO33 with the addition of semi-solid nanoparticles. This was attributed to the homogenous dispersion of PO33 nanoparticles facilitating the interaction between the nanoparticles and the starch.

### 3.7. Mechanical Properties of Starch–PO Films

**Figure 8:**
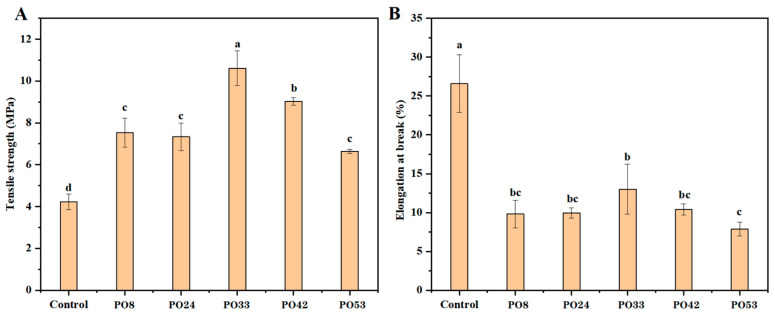
(**A**) TS and (**B**) EAB curves of starch–PO films containing POs with different melting points. Values with the same lower-case letter in a column are not statistically different (*p* > 0.05).

Figure 8 displays the TS and EAB values of the films with different melting points. The addition of PO nanoparticles increased the TS value. The high TS values may be explained by the reinforcement of interconnections between starch molecules by the palm oil nanoparticles, resulting in a stronger network structure. It has been reported that mango peel extract could enhance membrane networks through hydrogen bonding and hydrophobic interactions with gelatin molecules [31]. PO33 had the greatest TS value. This occurrence could be explained by the appropriate viscosity characteristics of semi-solid PO. The appropriate viscosity not only facilitated the formation of smaller oil droplets during the emulsification process, but also ensured the uniform dispersion of oil droplets. This distribution pattern helped PO nanoparticles to more effectively promote the formation of a tighter and more robust network between molecules. The TS values of films containing solid PO nanoparticles were lower than that of film containing semi-solid PO nanoparticles, which could also be explained from the perspective of the viscosity of PO. This phenomenon may alternatively be attributed to the higher crystallinity of solid POs. During film formation, crystallized oil particles interfered with the alignment and interconnection of molecules, making it difficult to form an ordered, dense network structure [32].

All of the starch–PO films’ EAB values were lower than the control film. The interaction force between the starch molecules was greatly increased by the addition of PO nanoparticles. This enhancement constituted an obstacle to the movement of starch molecules, thus hindering them to some extent and reducing their flexibility. The EAB value of PO33 was higher than the other four groups. This may be related to the phase state of the PO, the size of the PO nanoparticles and the thickness of starch–PO films. Semi-solid PO had the most moderate viscoelasticity, which facilitated the formation of PO nanoparticles of small diameter and uniform distribution. As shown in the CLSM image of Figure 5, the oil particles in PO8, PO24, PO42, and PO53 films were larger, introducing more obstacles that lead to more pronounced discontinuities in the film structure, thereby lowering the EAB value. Furthermore, an increase in film thickness could serve as an additional factor contributing to a decrease in the EAB value of the film. In general, the EAB of a film increases as the film thickness decreases. Compared with other films added with PO nanoparticles, PO33 had the lowest thickness value.

**Figure 9:**
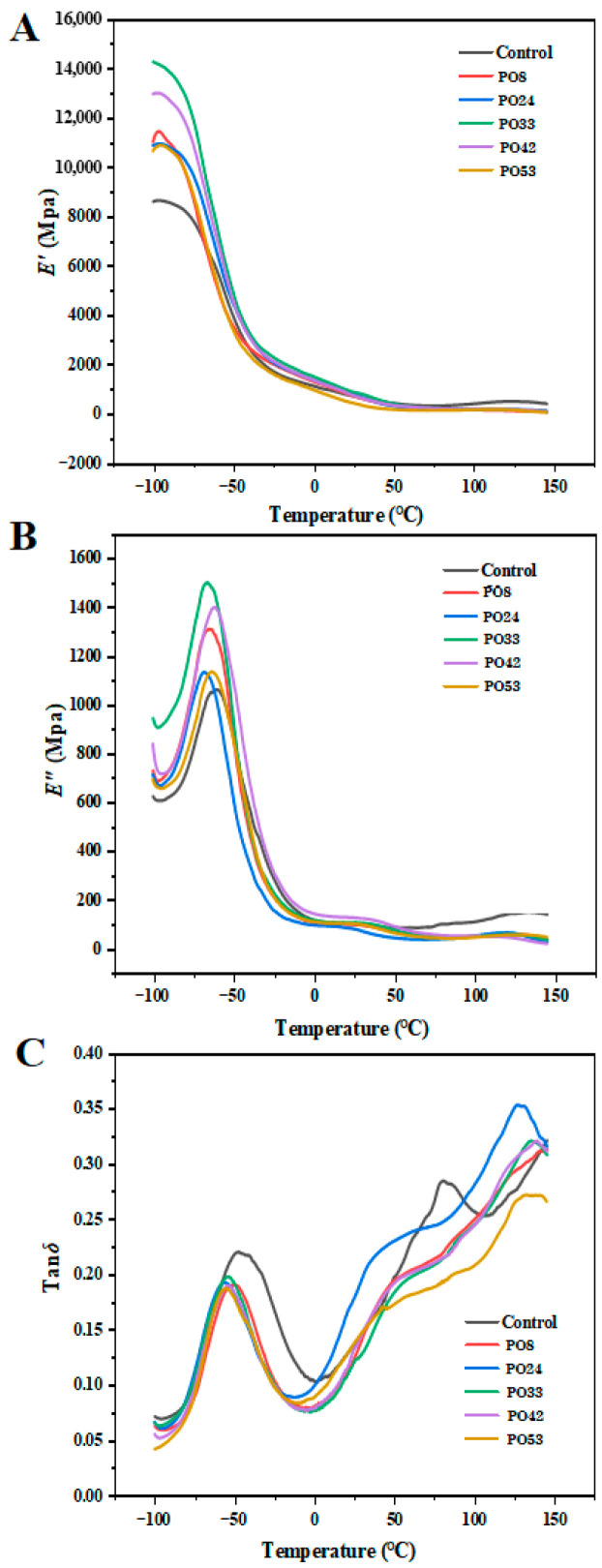
(**A**) E′, (**B**) E″ and (**C**) tan δ of starch–PO films containing POs with different melting points.

Figure 9 displays the storage modulus (E′), loss modulus (E″), and loss tangent (tan δ) of the starch–PO films. After PO nanoparticles were added, the films’ E′ and E″ values rose, indicating a stronger stiffness. The curve of PO33 was located at the top, indicating the highest values of E′ and E″, in agreement with its highest tensile strength value (Figure 8A). This outcome was likewise connected to the maximum crystallinity of PO33 (Figure 7). High crystallinity implied a more ordered arrangement of starch molecular chains, with enhanced intermolecular interactions, thereby rendering the film more rigid. The formation of a dense network structure and uniform oil droplet distribution in the films (as seen in Figure 5) also could limit the movement of the starch chains and increase film rigidity.

Two peaks in Figure 9C suggest that the substance had experienced two relaxing phases. The secondary transition of the glycerol-rich zone was associated with the initial relaxation temperature, which was around −50 °C [33]. As PO nanoparticles were added, the peak value of tan δ (−50 °C) shifted to the left, indicating that the addition of PO nanoparticles increased the compatibility between starch matrices. The second relaxation temperature (120–150 °C) may be related to the starch-rich phase in the films [34]. Compared with the control film, the tan δ peak shifted to the right after adding PO nanoparticles, which indicated that the film had better stiffness.

### 3.8. Thermal Properties of Films

**Figure 10:**
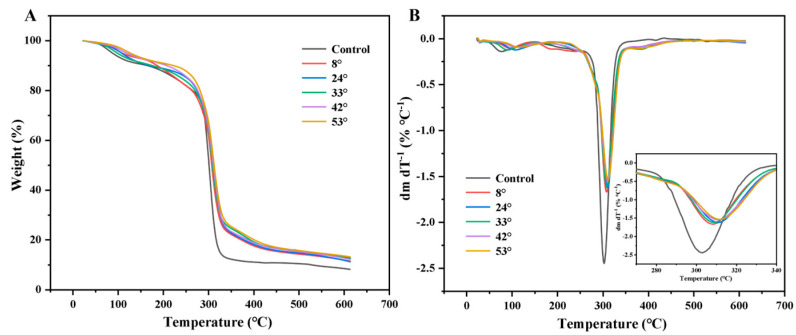
(**A**) TG and (**B**) DTG profiles of starch-PO films containing POs with different melting points.

Figure 10A shows that in the temperature range of 40–600 °C, the TG curves showed degradation peaks in the 182–320 °C region. This was primarily due to the thermal decomposition of starch–PO films, most of which could be explained by the breakdown of starch–PO films components and the volatilization of glycerol [35]. In Figure 10B, with the increase in the melting points of PO, the peak tips of the DTG curves of starch–PO films shifted to the right. The direction of this shift indicates changes in the thermal stability of films. A rightward shift signifies increased stability, while a leftward shift indicates decreased stability. The addition of PO nanoparticles resulted in improved thermal stability. This may be linked to the effective distribution of palm oil nanoparticles within the starch matrix, resulting in strong interactions with the matrix. Comparable thermal characteristics were observed for edible sugar palm starch–chitosan films fortified with extra virgin olive oil [30]. PO42 and PO53 showed the best thermal stability, followed by PO33, while PO8 and PO24 showed the least thermal stability. This may also be because solid PO nanoparticles provide better thermal stability than liquid PO emulsions. The thermal stability of starch composite films was reported to be enhanced by starch-palmitic acid complex nanoparticles [36].

## 4. Conclusions

In this study, emulsifier CAS and various physical states (liquid, semi-solid, and solid) of PO nanoparticles were incorporated into starch–PO films to enhance their properties. Results revealed that PO33 exhibited advantages in enhancing starch–PO film properties. Specifically, PO33 nanoparticles exhibited the smallest average diameter in the nanoemulsion system, gave starch–PO film an extremely smooth and homogeneous appearance, achieved optimal dispersion of the PO and promoted a high degree of crystallization within starch–PO films. This unique microstructure manifested in its macroscopic properties, exhibiting optimal water barrier performance, tensile strength, and film stiffness for PO33. In contrast, although the incorporation of liquid PO nanoparticles (PO8 and PO24) also improved starch–PO properties, the effect was not as significant as that of semi-solid PO nanoparticles. The incorporation of solid PO nanoparticles (PO42 and PO53), while providing better thermal stability to the films, was not as effective as the semi-solid PO nanoparticles in optimizing mechanical strength and water barrier properties.

This study shows that of the three states of nanoparticles, elastic semi-solid PO nanoparticles performed the best in improving the properties of starch–PO films. This study also introduces a novel idea that the use of elastic nanoparticles in a soft–hard equilibrium state is expected to lead to the development of better thin films.

## Figures and Tables

**Table 1:** Compositions of different starch–PO films.

Sample	10.0 wt% CS (g)	Nanoemulsions (PO = 1.0%, CAS = 0.25%) (g)	Water (g)	Glycerol (g)
control	25.0	0.0	25.0	0.7
PO8	25.0	25.0	0.0	0.7
PO24	25.0	25.0	0.0	0.7
PO33	25.0	25.0	0.0	0.7
PO42	25.0	25.0	0.0	0.7
PO53	25.0	25.0	0.0	0.7

**Table 2:** Average diameter and polydispersity index (Ð) of PO nanoemulsions.

PO Nanoemulsions	Average Diameter (nm)	Polydispersity Index (Ð)
PO8	374.5 ± 27.3 ^c^	0.510 ± 0.040 ^b^
PO24	447.8 ± 22.4 ^b^	0.508 ± 0.016 ^b^
PO33	346.5 ± 17.2 ^c^	0.434 ± 0.063 ^b^
PO42	524.0 ± 34.4 ^a^	0.771 ± 0.067 ^a^
PO53	573.3 ± 39.1 ^a^	0.843 ± 0.052 ^a^

Values with the same lower-case letter in a column are not statistically different (*p* > 0.05).

**Table 3:** Color parameters (L^*^, a^*^, b^*^) and total color difference (Δ*E*) of starch–PO films with different melting points.

Films	L^*^	a^*^	b^*^	Δ*E*
Control	53.93 ± 0.25 ^b^	1.81 ± 0.40 ^ab^	2.24 ± 0.33 ^b^	0
PO8	54.02 ± 0.54 ^b^	2.16 ± 0.10 ^ab^	2.34 ± 0.07 ^b^	0.56 ± 0.12 ^d^
PO24	54.54 ± 0.37 ^ab^	1.49 ± 0.55 ^ab^	3.37 ± 0.28 ^a^	1.33 ± 0.05 ^b^
PO33	52.84 ± 0.43 ^c^	2.29 ± 0.38 ^a^	3.54 ± 0.52 ^a^	1.74 ± 0.06 ^a^
PO42	54.72 ± 0.38 ^a^	1.72 ± 0.68 ^ab^	2.68 ± 0.41 ^b^	0.92 ± 0.56 ^c^
PO53	53.23 ± 0.12 ^c^	1.22 ± 0.59 ^b^	2.34 ± 0.39 ^b^	0.93 ± 0.13 ^c^

Values with the same lower-case letter in a column are not statistically different (*p* > 0.05).

**Table 4:** Basic properties of starch–PO films containing POs with different melting points.

Starch–PO Films	Thickness (mm)	Water Vapor Permeability (WVP) (1012 g·cm/cm^2^·s·Pa)	Moisture Content (%)	Total Soluble Matter Content (%)
control	0.143 ± 0.002 ^e^	3.871 ± 0.04 ^a^	15.58 ± 0.58 ^a^	31.22 ± 0.21 ^a^
PO8	0.190 ± 0.010 ^c^	3.403 ± 0.08 ^b^	14.64 ± 1.38 ^ab^	28.18 ± 0.14 ^b^
PO24	0.201 ± 0.002 ^b^	3.070 ± 0.08 ^c^	13.03 ± 0.37 ^bc^	29.54 ± 1.60 ^ab^
PO33	0.175 ± 0.003 ^d^	2.861 ± 0.07 ^d^	12.27 ± 0.28 ^c^	27.88 ± 0.29 ^b^
PO42	0.193 ± 0.004 ^c^	3.206 ± 0.06 ^c^	13.02 ± 1.43 ^bc^	28.16 ± 0.39 ^b^
PO53	0.225 ± 0.002 ^a^	3.708 ± 0.08 ^a^	14.52 ± 0.31 ^ab^	30.64 ± 1.40 ^a^

Values with the same lower-case letter in a column are not statistically different (*p* > 0.05).

## Data Availability

The original contributions presented in this study are included in the article. Further inquiries can be directed to the corresponding author.
